# Supplementary material for: Biochemical and functional characterization of the p.A165T missense variant of mitochondrial amidoxime-reducing component 1
Source: J Biol Chem. 2024 May 7;300(6):107353. doi: 10.1016/j.jbc.2024.107353 (PMC11190489; doi:10.1016/j.jbc.2024.107353)

Supporting information

Supplemental Figure 1.

Comparison of N-reductive activity in HepG2 (mARC1 C273A) with HepG2 (mARC1 C273A, MARC2 KO) cells

A. Benzamidine (BA) generated by the four cell lines in response to the treatment with various concentrations of BAO.

B. L-Arginine generated by the four cell lines in response to the treatment with various concentrations of NOHA.

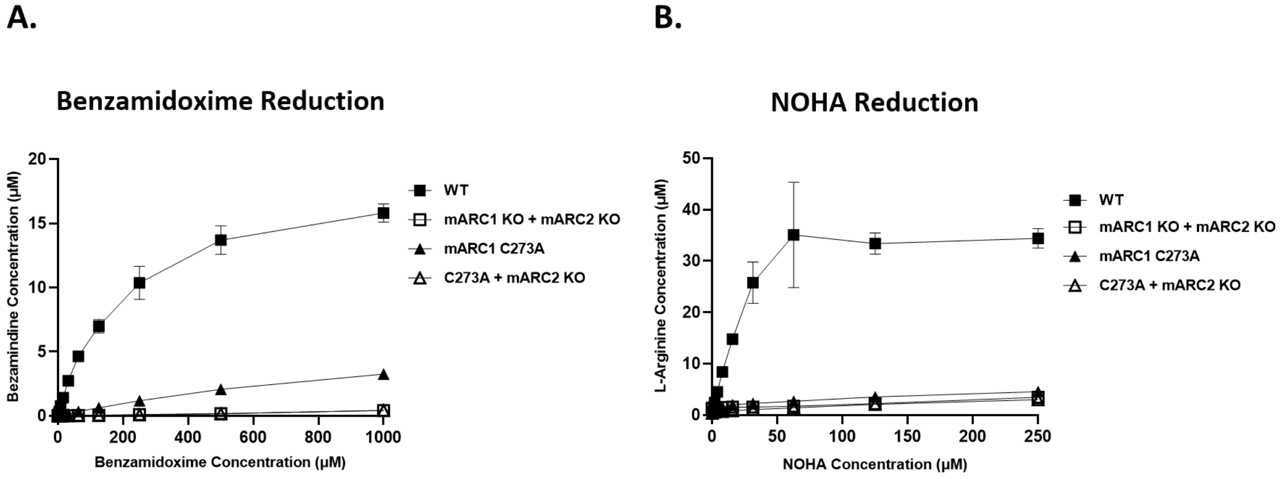

Supplement: Supplemental Figure 1 [file mmc1.pdf]
